# Risk for Asymptomatic Household Transmission of *Clostridioides difficile* Infection Associated with Recently Hospitalized Family Members

**DOI:** 10.3201/eid2805.212023

**Published:** 2022-05

**Authors:** Aaron C. Miller, Alan T. Arakkal, Daniel K. Sewell, Alberto M. Segre, Sriram V. Pemmaraju, Philip M. Polgreen, CDC MInD-Healthcare Group

**Affiliations:** University of Iowa, Iowa City, Iowa, USA

**Keywords:** C. difficile, Bacteria, enteric infections, antimicrobial resistance, Clostridioides difficile, hospitalization, asymptomatic infections, family transmission, risk factors, patient discharge, United States

## Abstract

We evaluated whether hospitalized patients without diagnosed *Clostridioides difficile* infection (CDI) increased the risk for CDI among their family members after discharge. We used 2001–2017 US insurance claims data to compare monthly CDI incidence between persons in households with and without a family member hospitalized in the previous 60 days. CDI incidence among insurance enrollees exposed to a recently hospitalized family member was 73% greater than enrollees not exposed, and incidence increased with length of hospitalization among family members. We identified a dose-response relationship between total days of within-household hospitalization and CDI incidence rate ratio. Compared with persons whose family members were hospitalized <1 day, the incidence rate ratio increased from 1.30 (95% CI 1.19–1.41) for 1–3 days of hospitalization to 2.45 (95% CI 1.66–3.60) for >30 days of hospitalization. Asymptomatic *C. difficile* carriers discharged from hospitals could be a major source of community-associated CDI cases.

*Clostridioides difficile* infection (CDI) is one of the most commonly occurring types of healthcare-associated infection and is predominately associated with hospitals (*1*,*2*). Thus, CDI-related investigations and interventions primarily have focused on hospital settings. More recently, reports of community-associated CDI cases, in which patients without a history of recent hospitalization are infected, have become more common (*3*,*4*). Although healthcare-associated CDI remains a considerable problem, more emphasis on community-associated CDI cases also is needed.

Risk factors for community-associated CDI are similar to risk factors for hospital-associated cases. For example, antimicrobial drug and proton-pump inhibitor (PPI) use increase the risk for community-associated CDI (*4*,*5*). For some community-associated CDI cases, exposure to healthcare settings beyond hospitalization, including clinics and emergency departments (*6*,*7*), are associated with an increased risk for CDI. However, for some CDI cases, no clear exposure to healthcare facilities can be identified. To find a source of *C. difficile* in community settings, other potential exposures have been proposed. Food is one such potential exposure, and *C. difficile* has been recovered from several different edible substances, including meat and vegetables (*8*,*9*). Pets have also been implicated (*10*). In addition, the possibility of household transmission of CDI between family members has been proposed, and having a symptomatic family member is a risk factor for CDI (*10*,*11*).

In addition to symptomatic CDI cases, patients with asymptomatic *C. difficile* colonization might contribute to transmission (*12*,*13*). In whole-genome sequencing studies, identifying epidemiologic links between symptomatic CDI among hospitalized patients has often been difficult (*14*,*15*), suggesting a potential role for asymptomatic *C. difficile* colonization. Asymptomatic colonized patients might contribute less to environmental contamination than symptomatic cases, but in sufficient numbers they could still play a role in *C. difficile* transmission in healthcare settings (*16*). Furthermore, if asymptomatically colonized patients contribute to *C. difficile* transmission within the hospital, then they could contribute to transmission in the community after they are discharged and especially could play a role in transmission among other household members. Finally, because hospitalized patients can remain asymptomatically colonized with *C. difficile* after discharge (*17*–*20*), this patient population could represent a large reservoir of CDI outside healthcare settings.

We investigated whether recently hospitalized patients increased the risk for CDI among household members in the period after discharge. Specifically, we were interested in the risk posed to household members by patients who are discharged without a CDI diagnosis and who are not diagnosed with CDI after discharge. If the risk for asymptomatic *C. difficile* colonization increases with length of stay, we hypothesized that the risk for CDI among household members should increase as a function of their recently hospitalized family members’ lengths of stay.

## Methods

### Data Source 

We constructed our study population from the US Commercial Claims and Medicare Supplemental datasets of IBM MarketScan Research Databases (https://www.ibm.com) from 2001–2017. These databases contain employer-sponsored commercial insurance claims and Medicare supplemental claims for >195 million enrollees during the 17-year study period. This dataset represents one of the largest longitudinal administrative databases in the United States. The databases provided insurance claims for inpatient, outpatient, and emergency department encounters, along with outpatient medications, demographic characteristics, employment, and enrollment characteristics. We were able to link claims from multiple family members in the same enrollment plan by using a family identifier along with a variable indicating each enrollee’s relationship to the primary enrollee, which indicated spouse, child, or dependent.

### Study Population 

We restricted our study population to enrolled households in which >2 family members could be identified on the same insurance plan. Our analysis was based on monthly CDI incidence, so we restricted our study population to those enrollees that were continuously enrolled for an entire month. We used code 008.45 from International Classification of Diseases, 9th Revision (ICD-9), and codes A04.7, A04.71, and A04.72 from the International Classification of Diseases, 10th Revision, Clinical Modification (ICD-10-CM), to identify CDI cases in outpatient and inpatient settings. To eliminate recurrent infections or subsequent care for the same infection, we focused on CDI cases in which the patient had no prior CDI diagnosis <60 days prior to the index month.

To isolate the potential effect of asymptomatic household transmission attributable to a prior hospitalization, we applied 2 additional restrictions to remove potential symptomatic exposures that might confound our results. First, we restricted our analysis to only enrollees that did not have a family member with CDI diagnosed in the period <60 days prior to the index month. Second, we restricted our analysis to those enrollees who were not hospitalized themselves <60 days prior to the index month.

### Analysis 

We compared the monthly incidence of CDI between persons in households where another family member had been recently hospitalized and discharged, <60 days prior to the index month, to those without recently hospitalized family members. We used a regression model to stratify enrollees into monthly enrollment strata based on the year and month, along with other demographic and patient characteristics, such as age, sex, prior antimicrobial drug use, PPI use, presence of an infant <2 years of age in the household, and exposure to a recently hospitalized family member. We then estimated the CDI incidence within each monthly enrollment strata, as a function of these various characteristics (Appendix).

We separated enrollees into categories for ages 0–17, 18–40, 41–65, and >65 years. We also categorized antimicrobial drugs into separate risk strata for high-CDI-risk antibiotics (clindamycin, fluoroquinolones, cephalosporins, carbapenems, ampicillin/sulbactam, pipercillin/tazobactam, and later-generation cephalosporins) or low-CDI-risk antibiotics (penicillin, macrolides, sulfonamides, trimethoprim, tetracyclines, and first-generation cephalosporins). We identified patients taking 1 of the following PPIs within 30 days before the CDI index date: omeprazole, esomeprazole, lansoprazole, rabeprazole, pantoprazole, dexlansoprazole, and omeprazole with sodium bicarbonate. We included an indicator for the presence of an infant <2 years old in the household because higher colonization rates have been found in infants (*17*,*21*).

#### Quantifying Exposure to Recently Hospitalized Family Members 

We evaluated the effect of exposure to a recently hospitalized family member in 2 ways. First, we defined a single dichotomous stratification based on whether any other family member spent time in the hospital <60 days prior to the index month. We then analyzed the incidence rate ratio (IRR) of CDI associated with exposure to a recently hospitalized family member. Second, we investigated whether a dose-response relationship existed between risk for CDI and the total amount of time that recently hospitalized family members spent in the hospital <60 days prior to the index month by computing the total days of within-family hospitalization. Specifically, we summed the lengths of stay across recently hospitalized family members’ inpatient stays that overlapped the previous 60-day exposure window. For example, a case-patient with 2 family members discharged in the prior 60 days, 1 with a length of stay of 2 days and the other 3 days, would have 5 total days of within-family hospitalization (Appendix Figure 1). Finally, we sorted total days of within-family hospitalization into categories of 0, 1–3, 4–10, 11–20, 21–30, and >30 days by using 0 days (i.e., no hospitalization or a hospitalization of <1 day) of prior exposure as the reference.

#### Statistical Approach 

We started by computing monthly CDI incidence for each of the patient characteristics used to define the various strata we described. We then estimated IRRs for the various patient strata while accounting for potential confounding effects by using a log-linear regression model, along with a quasi-Poisson distribution to account for overdispersion. Specifically, we estimated the mean CDI incidence in each monthly enrollment strata as a function of the binary criteria that define a stratum (Appendix). Of note, this approach and study population previously have been used to estimate the risk for secondary CDI infections among family members in household settings (*11*).

#### Sensitivity Analyses 

We conducted 2 sensitivity analyses. First, we evaluated whether underlying susceptibility at a household level might confound our results. For example, households with family members more susceptible to CDI also could be more likely to have longer or more frequent hospitalizations (Appendix Figure 2). To evaluate this effect, we analyzed 2 models in which we reversed the temporal order and evaluated whether CDI risk is associated with future hospitalizations in a family (Appendix).

Second, we explored the time window used to define prior exposures. Specifically, we considered a 90-day exposure window before index CDI events to compute total days of within household exposure and prior exposure to antimicrobial drugs.

## Results

We identified a total of 142,125,247 enrollees with >2 family members enrolled in the same insurance plan for an entire month (Table 1), which resulted in just over 5.1 billion enrollment months that we could observe over the study period. Most (53.2%) households contained >4 persons in the same insurance plan. We identified a total of 224,818 CDI cases across 194,424 enrollees; 55.9% of cases occurred among female enrollees and 74.6% among enrollees >40 years of age. Of all CDI cases, 6,575 cases represented a possible *C. difficile* transmission that occurred within 60 days after hospitalization of a family member. After we removed enrollees who were exposed to a family member with diagnosed CDI or who were hospitalized themselves, 164,650 CDI cases remained, of which 3,871 represented a potential asymptomatic *C. difficile* transmission from a recently hospitalized family member.

**Table 1:** Baseline enrollment characteristics of families with multiple infected members using a 60-day exposure window in study of asymptomatic *Clostridioides difficile* transmission among household members, United States*

Characteristics	All enrollees. no. (%)	No. (%) episodes of index CDI diagnosis†	No. (%) cases of possible transmission after family member hospitalization
No. CDI cases	NA	224,818	6,575
No. enrollees	142,125,247 (100)	194,424 (100)	6,453 (100)
Age group at enrollment or CDI diagnosis, y			
0–17	47,733,847 (33.6)	19,719 (8.8)	547 (8.3)
18–40	46,634,859 (32.8)	37,259 (16.6)	1,156 (17.6)
41–65	44,039,682 (31.0)	103,430 (46.0)	1,822 (27.7)
>65	3,716,859 (2.6)	64,410 (28.6)	3,050 (46.4)
Sex			
M	70,485,475 (49.6)	99,133 (44.1)	2,798 (42.6)
F	71,639,772 (50.4)	125,685 (55.9)	3,777 (57.4)
Family size			
2	36,598,138 (25.8)	134,644 (59.9)	4,166 (63.4)
3	29,857,746 (21.0)	36,236 (16.1)	905 (13.8)
4	40,705,784 (28.6)	34,559 (15.4)	839 (12.8)
5	21,536,725 (15.2)	13,517 (6.0)	409 (6.2)
>5	13,426,854 (9.4)	5,862 (2.6)	256 (3.9)

*CDI, *Clostridioides difficile* infection; NA, not applicable. †Events occurring >60 days before another episode.

We calculated CDI incidence rates of cases per 100,000 enrollment months and unadjusted IRRs by the various demographic and exposure groups (Table 2). Consistent with established CDI risk factors, we found CDI incidence was greater among female persons; persons >40 years of age, especially persons >65 years of age; persons with exposure to low-CDI-risk and high-CDI-risk antibiotics; and persons taking PPIs. Overall, the CDI incidence was ≈73% greater (IRR 1.73) among persons exposed to a recently hospitalized family member (incidence of 5.56 cases/100,000 enrollment months) than among persons who were not exposed to recently a hospitalized family member (incidence of 3.22 cases/100,000 enrollment months). At a bivariate level across nearly all enrollment characteristics, the CDI incidence rate was greater among enrollees in households with recently hospitalized family members (Table 3). CDI incidence increased monotonically across the various levels of within-household hospitalization from 3.22 cases/100,000 enrollment months for 0 days of within-household hospitalization to 8.73 cases/100,000 enrollment months for >30 total days of within-household hospitalization.

**Table 2:** Bivariate comparisons of unadjusted incidence rates and incidence rate ratios for infection incidence across various patient strata using a 60-day exposure window in study of asymptomatic *Clostridioides difficile* transmission among household members, United States*

Variable	Exposed to previously hospitalized family member <60 days				Not exposed to previously hospitalized family member <60 days			Unadjusted IRR
	CDI cases	Total enrollee months	CDI incidence†		CDI cases	Total enrollee months	CDI incidence†	
Overall	3,871	69,675,026	5.56		160,779	4,998,101,178	3.22	1.73
Age group, y								
0–17	317	24,432,280	1.30		15,615	1,445,786,086	1.08	1.20
18–40	567	19,978,891	2.84		29,718	1,427,785,479	2.08	1.37
41–65	1,193	19,281,059	6.19		74,803	1,868,106,655	4.00	1.55
>65	1,794	5,982,798	29.99		40,643	256,422,958	15.85	1.89
Sex								
M	1,698	37,945,564	4.47		67,378	2,488,714,427	2.71	1.65
F	2,173	31,729,463	6.85		93,401	2,509,386,752	3.72	1.84
Outpatient antimicrobial drug use within 60 days								
None	2,419	63,230,032	3.83		100,792	4,575,861,567	2.20	1.74
Low-risk drugs	292	2,979,748	9.80		11,944	201,200,918	5.94	1.65
High-risk drugs	1,160	3,465,248	33.48		48,043	221,038,693	21.74	1.54
PPI use within 30 days								
N	3,477	68,273,806	5.09		146,185	4,913,346,960	2.98	1.71
Y	394	1,401,221	28.12		14,594	84,754,218	17.22	1.63
Infant age <2 y in family									
N	3,489	48,618,765	7.18		151,291	4,597,497,625	3.29	2.18	
Y	382	21,056,262	1.81		9,488	400,603,553	2.37	0.76	

*IRRs compare CDI incidence among persons exposed to a family member previously hospitalized for >1 d relative incidence for to those not exposed to a previously hospitalized family member. The overall incidence rate ratio among those exposed to a previously hospitalized family member relative to those unexposed was 1.73 and was >1 across all strata. CDI, *Clostridioides difficile* infection; IRR, incidence rate ratio; PPI, proton-pump inhibitor. †Cases per 100,000 enrollee months.

**Table 3:** Number of cases and enrollee-months in each exposure bin for total days of household-hospitalization using a 60-day exposure window in study of asymptomatic *Clostridioides difficile* transmission among household members, United States*

No. days family members spent hospitalized	60-day exposure window		
	No. CDI cases	Total enrollment months	Incidence†
0	160,267	4,980,648,694	3.22
1–3	2,336	52,798,719	4.42
4–10	1,519	27,457,461	5.53
11–20	315	4,338,929	7.26
21–30	107	1,317,610	8.12
>30	106	1,214,792	8.73

*CDI, *Clostridioides difficile* infection. †Cases per 100,000 enrollment months.

For stratified regression analyses, we divided enrollees into 357,348 enrollment-month strata based on different combinations of demographics, enrollment characteristics, and risk factors (Table 4). For each within-household hospitalization exposure group, we computed IRRs relative to the baseline group in which family members spent <1 day in the hospital during the previous 60 days. Compared with enrollees whose family members spent <1 day in the hospital, the IRR of CDI continuously increased across the exposure bins from 1.30 (95% CI 1.19–1.41) for persons with 1–3 days of within-family hospitalization up to 2.45 (95% CI 1.66–3.60) for those with >30 days of within-family hospitalization.

**Table 4:** Results of regression analysis of incidence rate ratio for *Clostridioides difficile* infection using quasi-Poisson model and 60-day exposure window in study of asymptomatic *C. difficile* transmission among household members, United States*

Variable	IRR (95% CI)
No. days member was hospitalized within 60 d	
0	Referent
1–3	1.30 (1.19–1.41)
4–10	1.46 (1.32–1.62)
11–20	1.79 (1.43–2.23)
21–30	2.17 (1.48–3.18)
>30	2.45 (1.66–3.60)
Age group, y	
0–17	Referent
18–40	1.71 (1.65–1.78)
41–65	2.97 (2.86–3.08)
>65	9.32 (8.92–9.73)
Sex	
M	Referent
F	1.30 (1.28–1.33)
Outpatient antimicrobial drug use within 60 d	
None	Referent
Low-risk drugs	2.69 (2.59–2.79)
High-risk drugs	8.83 (8.63–9.03)
PPI use within 30 d	2.23 (2.15–2.30)
Infant <2 y in family	1.51 (1.44–1.58)

*Models were adjusted for year, month, and family size. Regression models included an offset for number of enrollment months. Because family hospitalization exposure group was followed for 60 days to identify secondary *Clostridioides difficile* infection, the length of their enrollment period is 60 days. For the unexposed group, the length of enrollment was the length of a given month. IRR, incident rate ratio; PPI, proton-pump inhibitor.

Known CDI risk factors also were associated with greater incidence. Antimicrobial drug exposure was associated with an increased CDI incidence rate; for low-CDI-risk antibiotics the IRR was 2.69 (95% CI 2.59–2.79), and for high-CDI-risk antibiotics IRR was 8.83 (95% CI 8.63–9.03). PPI usage was also associated with statistically significant CDI incidence, an IRR of 2.23 (95% CI 2.15–2.30). CDI incidence increased with age; relative to ages 0–17 years the IRR continuously increased from 1.71 (95% CI 1.65–1.78) for ages 18–40 years to 9.32 (95% CI 8.92–9.73) for ages >65 years. Female persons had a higher incidence compared with male persons (IRR 1.30, 95% CI 1.28–1.33). Households with an infant also had a higher CDI incidence than those without (IRR 1.51, 95% CI 1.44–1.58).

We performed a sensitivity analysis to determine whether our results were confounded by household-level susceptibility (Appendix Table 1). When we reversed the temporal order of hospital exposure, we found little evidence that our primary results can be explained by confounding due to CDI susceptibility among family members. The point estimates for our primary dose-response curve remained relatively unchanged and were considerably larger than the effect estimates associated with future hospital visits among family members.

As a second sensitivity analysis, we considered a 90-day exposure window for capturing recently hospitalized family members (Appendix Tables 2–4). In general, the results of the analysis using a 90-day exposure window were consistent with the 60-day window, and we noted a similar dose-response relationship between the total days of within-household hospitalization among recently hospitalized family members and risk for CDI. However, the magnitude of some of the point estimates was slightly attenuated using the 90-day window compared with the 60-day window. For example, the IRR for the 1–3 day within-family hospitalization category was 1.24 for the 90-day window, compared with 1.30 for the 60-day window. However, the CIs for both sets of analyses overlapped the point estimates of the other.

## Discussion

In this study, we found that persons exposed to recently hospitalized family members were at substantially increased risk for CDI within 60 days after the family member’s hospital discharge. Furthermore, CDI risk among family members increased as total days of within-household hospitalization increased. Because CDI was not diagnosed in recently hospitalized and discharged family members during or after their hospitalization, and because persons in our analysis were not hospitalized themselves, the increased risk could be attributable to asymptomatic *C. difficile* colonization at the time of hospital discharge in the hospitalized family member.

We also conducted several sensitivity analyses. First, to evaluate whether household confounding because of greater hospitalization in more susceptible family members could explain our findings, we reversed the temporal ordering of hospital exposure and found that incorporating future hospitalizations did not attenuate our primary effect estimates. This finding reinforces our primary hypothesis that the increased risk we observed is attributable to transmission from family members who become asymptomatically colonized during a prior hospital stay. Second, we used a 90-day exposure window and found consistent results but the dose-response effect appeared slightly attenuated. This finding could suggest that household exposures occurring >60 days in the past might convey minimal risk.

Our results have several implications. First, we provide further support for the role of asymptomatic *C. difficile* carriers in bacterial transmission. Second, we identify a previously underappreciated potential CDI reservoir outside healthcare settings that could support the spread of community-associated *C. difficile*. Finally, our results suggest that, if patients who are asymptomatically colonized during a hospital stay contribute to transmission in the community, not all CDI cases attributable to hospital exposure can be directly identified based on hospital discharge records.

In hospital settings, patients asymptomatically colonized with *C. difficile* are increasingly viewed as a major contributor to CDI spread (*12*,*13*). Indeed, asymptomatic *C. difficile* transmission has been posited as an explanation for the missing epidemiologic links in whole-genome sequencing studies (*14*). Asymptomatic *C. difficile* colonization among hospitalized patients is not uncommon (*12*,*17*–*20*). For example, a meta-analysis found that ≈10% of hospitalized patients in North America become colonized (*20*). In addition, the likelihood of colonization increases with longer hospital stays (*17*), as well as the use of chemotherapy (*22*), PPIs or H2 blockers (*22*), and steroids (*23*). Furthermore, colonization likely persists for some time after discharge. For example, prior hospitalization, even 6 months in the past, has been found to be a risk factor for colonization at hospital admission (*18*). Because asymptomatically colonized patients can contaminate the environment and *C. difficile* spores are resistant to many cleaning solutions, household environments could feasibly lead to both symptomatic and asymptomatic CDI in family members.

Despite the increase in community-acquired CDI, relatively little research has focused on the household setting. Instead, most efforts to find the exposure sources for community-associated CDI have focused on healthcare settings outside hospitals, such as outpatient clinics and emergency departments (*6*,*7*), and nonhealthcare sources such as food (*8*), household pets (*10*), and even exposure to the agricultural industry (*24*). A few relatively small studies (*10*,*25*) and 1 large study (*11*) did identify potential secondary *C. difficile* transmission from symptomatic cases among household members. Thus far, however, few studies, except studies focusing on newborns, have questioned the role of asymptomatic carriers in household settings. Because infants frequently are colonized with *C. difficile* in their first several months of life, our findings and those from other studies that exposure to infants is potential risk factor for community-associated *C. difficile* (*17*,*21*) are not surprising.

Household transmission has been documented for other gastrointestinal infections, including rotavirus, norovirus, and *Giardia* (*26*–*30*). In addition, household transmission has been documented for another major healthcare-associated infection, methicillin-resistant *Staphylococcus aureus* (*31*,*32*). For at least some of these pathogens, asymptomatic or minimally symptomatic cases contribute to disease transmission. Of note, transmission of methicillin-resistant *S. aureus*, like *C. difficile,* was first thought to be almost exclusively confined to hospital settings; awareness of spread in community settings emerged later. Close household contact can also contribute to the spread of other fecal–oral pathogens, such as rotavirus and norovirus (*27*), via environmental contamination, providing further support for the plausibility of household spread of *C. difficile*.

In addition to providing support for the contribution of asymptomatic *C. difficile* colonization to household transmission, our results also might have implications for future *C. difficile* surveillance and intervention-based investigations. Prior investigations have shown that cases of symptomatic hospital-associated CDI often do not appear until after a patient is discharged (*33*) and that some of those cases might generate additional symptomatic cases among family members (*11*). However, our results raise the policy question of whether secondary symptomatic cases among household members should be considered when measuring the broader costs of healthcare-associated infections, especially those that have a reasonable epidemiologic link (e.g., using genotyping) with discharged patients who are asymptomatically colonized. Our results clearly suggest that hospital-based interventions to control both symptomatic and asymptomatic *C. difficile* transmission can help reduce spread in the community. Measures based on standard surveillance efforts might also underestimate the full effectiveness of hospital-based infection and antimicrobial stewardship interventions because those measures might not capture potential, positive downstream effects in the community.

One limitation of our study is that we cannot directly identify the exact point of exposure where *C. difficile* transmission might have occurred. Exposure could have occurred in a household setting after a family member was discharged from the hospital; alternatively, a family member might have become colonized while visiting another family member in the hospital. However, several reasons exist to suspect that family members visiting the hospital are unlikely to fully explain our observed effect. First, healthcare workers often have lower colonization rates than discharged patients (*17*). Second, visitors and visiting hours often are limited or restricted and only represent a small portion of a patient’s total length of stay. Third, we did not count persons as exposed in our analysis when their corresponding CDI index date occurred before their family members were discharged from the hospital; we only consider exposure to a recently hospitalized family member after discharge occurred. Thus, if visiting the hospital were the primary mechanism driving our results, our analytical method would be greatly biased toward the null.

Another limitation of our study is that we depended on insurance claims data and diagnostic codes to identify CDI events. We did not have access to laboratory test results to confirm CDI diagnoses, nor did we have access to genetic data to confirm whether subsequent CDI cases in family members were genetically related. We also could not observe or confirm that household contact actually occurred in the assumed household setting; family members could be residing in different locations even if they were enrolled in the same insurance plan. Finally, our data might not capture all family members residing in a single location. We only had access to information for family members that are actively enrolled in the same insurance plan, and family members in the same household are often enrolled in different plans. Despite these limitations, our results demonstrate the importance of considering asymptomatic carriers in spread of CDI in household settings. 

In conclusion, because patients are frequently colonized with *C. difficile* during hospitalization and at discharge, and because ≈25 million persons each year have overnight hospital stays in the United States alone (*34*), patients recently discharged from hospitals could be spreading *C. difficile* outside hospital settings. Asymptomatic *C. difficile* carriers discharged from hospitals could be a major source of community-associated CDI cases and should be considered during surveillance and intervention-based investigations.

Appendix Additional information on asymptomatic household transmission of *Clostridioides difficile* infection associated with recently hospitalized family members.
