# Supplementary material for: Risk for Asymptomatic Household Transmission of Clostridioides difficile Infection Associated with Recently Hospitalized Family Members
Source: Emerg Infect Dis. 2022 May;28(5):932–9. doi: 10.3201/eid2805.212023 (PMC9045444; doi:10.3201/eid2805.212023)
Supplement: Appendix — Additional information on asymptomatic household transmission of Clostridioides difficile infection associated with recently hospitalized family members. [file 21-2023-Techapp-s1.pdf]

Article DOI: https://doi.org/10.3201/eid2805.212023

Risk for Asymptomatic Household Transmission of *Clostridioides difficile* Infection Associated with Recently Hospitalized Family Members

Appendix

Additional Methods

Data Construction and Statistical Model

Our analysis was conducted at an aggregated monthly incidence level, in which we aggregated cases of *Clostridioides difficile* infection (CDI) into monthly-enrollment strata. We computed the outcome variable as number of CDI cases in a monthly-enrollment stratum, where we defined the strata by the various covariates used in our analysis. For example, if the dependent variables were year, month, sex, age, and prior household exposure, 1 row of data might be defined by the stratum for 2015, December, female sex, age 18–40 years, and family members hospitalized for 5–10 days. In this stratum, the dependent variable would be the count of the number of enrollees who had CDI, and the independent variables would be indicators for each of the categorical features characterizing the stratum (year, month, sex, and age group). We also included an offset to control for the overall size of the enrolled population in the stratum. This stratification resulted in 357,348 enrollment-month-strata, defined by the distinct combinations of covariates. Multiple enrollees could count toward the CDI incidence in each stratum, but a single enrollee would be counted only in as many different strata as the number of months in which they satisfied enrollment criteria.

To be included in the model, an enrollee could not have been hospitalized ≤60 days prior to the index month. For each month that an enrollee appeared in the claims data, we checked to determine if the enrollee was hospitalized in the period ≤60 days prior to the start of the particular monthly-enrollment stratum. For patients with diagnosed CDI, we used the 60 days

before the admission date of the visit where they received a CDI diagnosis. Thus, if an enrollee was hospitalized, they would be removed from our analysis and the corresponding monthly enrollment strata for months where the start of the month occurred (i.e., <60-days after the discharge date of the prior hospitalization). Furthermore, they would re-enter the analysis once >60 days passed between the start of the monthly enrollment period and the discharge date of their previous hospitalization.

Similarly, we excluded enrollees in a household where another family member was diagnosed with CDI in the period ≤60 days prior to the index month. For enrollees with diagnosed CDI, we used the date of their CDI diagnosis, or discharge date if diagnosis occurred during an inpatient hospital stay, to define the 60-day "washout" period. Thus, we excluded an enrollee from a given enrollment-month strata if they had a CDI diagnosis ≤60 days prior to the start of the enrollment month, and we would re-include them in subsequent enrollment-month strata after 60 days passed between the start of the enrollment month and the discharge date, or diagnosis date for outpatient visits, of the previous CDI diagnosis. Moreover, because we also excluded enrollees in households where another family member was diagnosed with CDI in the period ≤60 days prior to the index month, once 1 family member was diagnosed, we excluded all other family members in the household from our analysis for the months where the start date of a month was ≤60 days of the corresponding CDI diagnosis. Thus, in any given 120-day period, or 180-day period for our sensitivity analysis (described below), we included only 1 CDI case from a given household in our analysis.

After constructing enrollment strata, we used the following model to estimate the incidence rate ratio (IRR) associated with CDI:

$$log(MeanCDI_j) = \alpha + X_j\beta + log\ (enrollment_j)$$

where for stratum j, $MeanCDI_j$ is the expected count of the number of CDI cases in j, X_j are the set of indicators used to define j (e.g., prior family hospitalization days, age bin, sex, high-risk antibiotics, etc.), and $enrollment_j$ is an offset term used to control for the enrollment size of stratum j. We used a quasi-Poisson distribution to account for overdispersion.

Sensitivity Analysis for Confounding Due to Household Susceptibility

Persons living in a household in which other family members have a higher susceptibility to CDI might also be at increased risk for CDI because such family members are more susceptible and, thus, more likely to be colonized in general. In addition, family members who are more susceptible to CDI might tend to have more frequent, or longer, hospital stays, contributing to a greater level of household-hospital exposure. Thus, our observed relationship between the time other family members in a household spent hospitalized and the risk for CDI might not reflect asymptomatic transmission attributable to prior hospitalization in a family member, but rather the confounding effect of having family members who are more susceptible to CDI. We devised a directed acyclic graph to visualize the relationship of the potential confounding effect and our hypothesized relationship (Appendix Figure 2).

To evaluate whether this potential confounding effect could explain our observed relationship, we performed a sensitivity analysis in which we reversed the temporal order between CDI cases and the hospitalizations in other family members. Specifically, we evaluated if future hospitalizations (i.e., in the 60 days after a given exposure window) were associated with increased risk for CDI. If the potential confounding relationship described above is true, we would expect that persons in households with family members having a greater underlying hospitalization risk (i.e., CDI susceptibility) would also have a greater CDI risk associated with family hospitalizations occurring in the future.

We considered 2 analyses to evaluate the effect of household CDI on future hospitalizations. First, we incorporated a single indicator into our model for persons with any family member hospitalized for any amount of time in the period $\leq$60 days after the index month. Second, we incorporated future family hospitalization exposure bins like those used in our primary analysis, but where we binned the number of days other family members were hospitalized in the future. We computed future household hospitalization bins in the same manner as described in our primary manuscript; however, we used hospitalizations that occurred in other family members in the 60 days after a given exposure month. Specifically, we summed all days that other family members were hospitalized in the 60 days after an exposure-month across all other family members who were hospitalized. We then binned these exposure counts into the same groups used in our primary analysis (i.e., 0 days, 1–3 days, 4–10 days, etc.). Finally, we evaluated whether either of these future hospitalization measures were associated

with monthly CDI incidence and if their inclusion into the model attenuated any of the observed relationships in our prior family hospitalization bins.

We summarized the primary effect estimates for our model from the main manuscript, the model where we added a single future hospitalization indicator, and the model where we incorporated multiple exposure bins for the duration of future hospitalization among family members (Appendix Table 1). From the 2 models, we saw that a slightly positive association does occur between future hospitalization among family members and CDI risk in a given exposure month. The model where we incorporated future hospitalization-exposure bins also demonstrated a slight dose-response relationship for days <30; although, the bins >21 days are not statistically significant and the bin for >30 days is associated with lower CDI risk. Each of the effect estimates for future hospitalization were much smaller in magnitude than the effects for prior hospitalization. Moreover, after incorporating the measures of future hospitalization into the model, we saw no real attenuation of the primary dose-response relationship with the prior hospitalization bins. In both models where we add future hospitalizations, we found the same general dose-response effect for prior family hospitalization which remains highly statistically significant. Thus, we do not find evidence that our primary dose-response effect estimates are confounded at a household level by family members more likely to be hospitalized in general.

Appendix Table 1. Sensitivity analysis for confounding due to household susceptibility and the effect of incorporating measures of future hospitalization among family members into the model for *Clostridioides difficile* infection risk associated with prior hospitalization among family members*

Variable	Incidence rate ratio (95% CI)		
	Primary model	Single future exposure bin	Multiple future exposure bins
No. days family members were hospitalized ≤60 d			
0	Referent	Referent	Referent
1–4	1.30 (1.19–1.41)	1.29 (1.20–1.39)	1.29 (1.21–1.37)
5–10	1.46 (1.32–1.62)	1.45 (1.33–1.59)	1.45 (1.34–1.56)
11–20	1.79 (1.43–2.23)	1.75 (1.44–2.13)	1.74 (1.47–2.06)
21–30	2.17 (1.48–3.18)	2.12 (1.52–2.96)	2.11 (1.58–2.81)
>30	2.45 (1.66–3.6)	2.38 (1.70–3.33)	2.37 (1.78–3.17)
No. days family members were hospitalized in next 60 d			
0	–	Referent	Referent
Any future hospitalization	–	1.14 (1.07–1.20)	–
1–4	–	–	1.09 (1.02–1.16)
5–10	–	–	1.20 (1.10–1.30)
11–20	–	–	1.24 (1.03–1.51)
21–30	–	–	1.32 (0.93–1.88)
>30	–	–	0.87 (0.51–1.47)
Age group, y			
0–17	Referent	Referent	Referent
18–40	1.71 (1.65–1.78)	1.71 (1.65–1.77)	1.71 (1.66–1.76)
41–65	2.97 (2.86–3.08)	2.97 (2.87–3.07)	2.97 (2.89–3.05)
>65	9.32 (8.92–9.73)	9.29 (8.95–9.65)	9.29 (9.00–9.60)
Sex			
M	Referent	Referent	Referent
F	1.30 (1.28–1.33)	1.30 (1.28–1.33)	1.30 (1.28–1.32)
Outpatient antimicrobial drug use ≤60 d			
None	Referent	Referent	Referent
Low-risk drugs	2.69 (2.59–2.79)	2.69 (2.60–2.78)	2.69 (2.61–2.76)
High-risk drugs	8.83 (8.63–9.03)	8.83 (8.65-9.00)	8.83 (8.68–8.98)
PPI use ≤30 d	2.23 (2.15–2.3)	2.22 (2.16–2.29)	2.22 (2.17–2.28)
Infant in family	1.51 (1.44–1.58)	1.50 (1.44–1.56)	1.50 (1.45–1.55)

*PPI, proton-pump inhibitor.

Appendix Table 2. Baseline enrollment characteristics for families with multiple members infected with *Clostridioides difficile* using a 90-day exposure window in a study of asymptomatic *C. difficile* transmission among household members*

Characteristics	All enrollees	Episodes of index CDI diagnosis ≥90 d of another episode	Possible transmission after family member hospitalization
No. CDI cases	NA	216,198	8,617
No. enrollees	142,125,247 (100)	194,396 (100)	8,482 (100)
Age group at enrollment or CDI diagnosis (years)			
0–17	47,733,847 (33.6)	19,058 (8.8)	791 (9.2)
18–40	46,634,859 (32.8)	35,960 (16.6)	1,516 (17.6)
41–65	44,039,682 (31.0)	99,581 (46.1)	2,377 (27.6)
>65	3,716,859 (2.6)	61,599 (28.5)	3,933 (45.6)
Sex			
M	70,485,475 (49.6)	95,595 (44.2)	3,736 (43.4)
F	71,639,772 (50.4)	120,603 (55.8)	4,881 (56.6)
Family Size			
2	36,598,138 (25.8)	129,292 (59.8)	5,405 (62.7)
3	29,857,746 (21.0)	34,886 (16.1)	1,213 (14.1)
4	40,705,784 (28.6)	33,322 (15.4)	1,128 (13.1)
5	21,536,725 (15.2)	13,049 (6.0)	536 (6.2)
>5	13,426,854 (9.4)	5,649 (2.6)	335 (3.9)

*Values represent no. (%). CDI, *Clostridioides difficile* infection; NA, not applicable.

Appendix Table 3. Bivariate comparisons of *Clostridioides difficile* incidence using a 90-day exposure window in a study of asymptomatic *C. difficile* transmission among household members*

	Exposed to family member who was hospitalized ≤90 d			Not exposed to family member who was hospitalized ≤90 d			
Variable	CDI cases	Total enrollee months	CDI incidence†	CDI cases	Total enrollee months	CDI incidence†	Unadjusted IRR
Overall	4,498	98,864,945	4.55	141,080	4,946,063,813	2.85	1.60
Age group, y							
0–17	426	34,948,896	1.22	14,333	1,430,683,172	1.00	1.22
18–40	713	28,337,246	2.52	27,277	1,412,440,836	1.93	1.31
41–65	1,430	27,180,485	5.26	66,975	1,852,483,156	3.62	1.45
>65	1,929	8,398,319	22.97	32,495	250,456,649	12.97	1.77
Sex							
M	1,979	54,062,482	3.66	58,233	2,463,233,320	2.36	1.55
F	2,519	44,802,463	5.62	82,847	2,482,830,493	3.34	1.68
Outpatient antimicrobial drug use ≤90 d							
None	2,538	86,762,924	2.93	78,650	4,395,042,891	1.79	1.64
Low-risk drugs	359	5,495,746	6.53	11,576	255,610,790	4.53	1.44
High-risk drugs	1,601	6,606,275	24.23	50,854	295,410,133	17.21	1.41
PPI use ≤30 d							
N	4,063	96,890,799	4.19	129,000	4,863,125,084	2.65	1.58
Y	435	1,974,146	22.03	12,080	82,938,729	14.56	1.51
Infant in Family							
N	3,992	68,579,807	5.82	132,577	4,562,295,268	2.91	2.00
Y	506	30,285,138	1.67	8,503	383,768,544	2.22	0.75

*CDI, *Clostridioides difficile* infection; IRR, incident rate ratio; PPI, proton-pump inhibitor.
†CDI incidence per 100,000 enrollee months.

Appendix Table 4. Analysis and incident rate ratios using quasi-Poisson models and a 90-day exposure window in a study of asymptomatic *Clostridioides difficile* transmission among household members*

Variable	Incidence rate ratio (95% CI)
No. days family member hospitalized ≤90 d	
0	Referent
1–3	1.24 (1.16–1.33)
4–10	1.39 (1.27–1.52)
11–20	1.60 (1.32–1.94)
21–30	1.80 (1.28–2.53)
>30	2.49 (1.85–3.36)
Age group, y	
0–17	Referent
18–40	1.73 (1.67–1.79)
41–65	2.91 (2.80–3.01)
>65	8.19 (7.85–8.54)
Sex	
M	Referent
F	1.3 (1.27–1.32)
Prior outpatient antimicrobial drug use ≤90 d	
None	Referent
Low-risk drugs	3.47 (3.35–3.60)
High-risk drugs	12.51 (12.24–12.78)
PPI use ≤30 d	2.00 (1.93–2.07)
Infant in family	1.51 (1.44–1.57)

*PPI, proton-pump inhibitor.

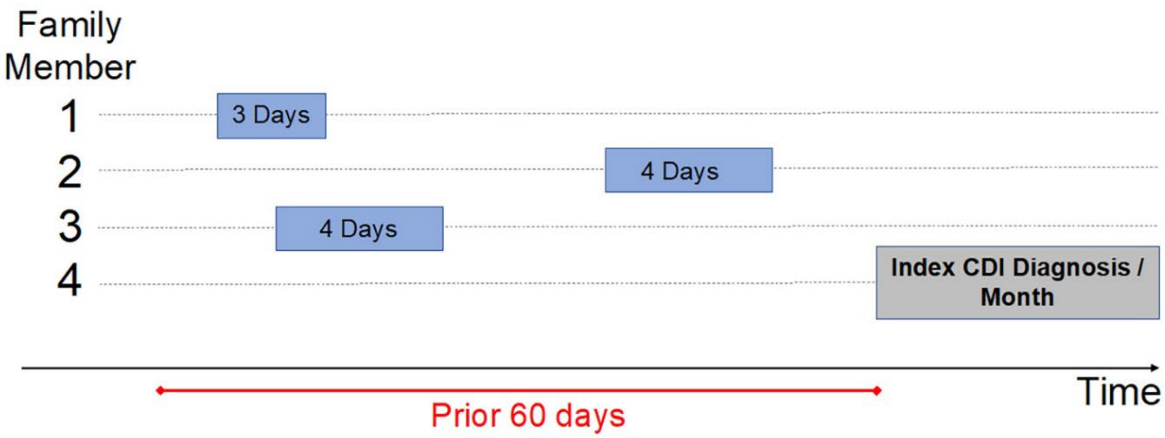

Appendix Figure 1. Example visualization of the approach used to compute total length of hospital exposure among enrollees in a study of asymptomatic *Clostridioides difficile* transmission among household members. For each person, the total exposure risk is represented as a sum across all prior hospitalizations in all other family members occurring <u><</u>60 days prior to the index month. In this example, family member 4 would have 11 days of total family hospital exposure relative to their index *C. difficile* diagnosis or enrollment month.

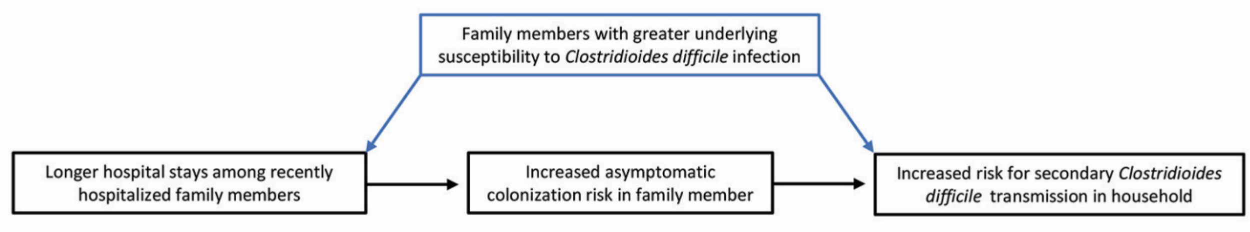

Appendix Figure 2. Directed acyclic graph of potential confounding effect in a study of asymptomatic *Clostridioides difficile* transmission among household members. Black indicates the hypothesized relationship; longer hospital stays among family members are associated with increased risk for asymptomatic colonization and subsequent transmission to family members in the household. Blue indicates a potential confounding bias; family members with greater underlying susceptibility to *C. difficile* infection might have longer or more frequent hospital stays and be more likely to transmit *C. difficile* to other family members.
